# Metabolic changes in cardiomyocytes during sepsis

**DOI:** 10.1186/cc13011

**Published:** 2013-09-20

**Authors:** James J Douglas, Keith R Walley

**Affiliations:** 1 Centre for Heart and Lung Innovation, St Paul's Hospital and University of British Columbia, Division of Critical Care Medicine, Vancouver, British Columbia V6Z 1Y6, Canada

## Abstract

Different types of shock induce distinct metabolic changes. The myocardium at rest utilizes free fatty acids as its primary energy source, a mechanism that changes to aerobic glycolysis during sepsis and is in contrast to hemorrhagic shock. The immune system also uses this mechanism, changing its substrate utilization to activate innate and adaptive cells. Cardiomyocytes share a number of features similar to antigen-presenting cells and may use this mechanism to augment the immune response at the reversible expense of cardiac function.

## 

Cardiomyocyte metabolism changes in response to different types of shock. Chew and colleagues [1] in the previous issue of *Critical Care* used microdialysis to measure metabolites within cardiac and skeletal muscle during endotoxemia. They found that endotoxemic shock induces metabolic changes in cardiac and skeletal muscle cells that do not occur during hemorrhagic shock. In particular, myocardial and skeletal muscle glucose concentrations were markedly decreased during endotoxemic shock. While lactate increased in both forms of shock, pyruvate levels also increased during endotoxemia, suggesting that elevated lactate levels were not due to anaerobic metabolism, in accord with the observations of Cunnion and colleagues [2]. Muscle pyruvate levels did not increase to the same extent during hemorrhagic shock, suggesting that increased lactate was linked to anaerobic metabolism occurring in this form of shock.

This study demonstrated novel findings on the metabolic differences between two pathological shock states and re-demonstrated the metabolic flexibility of the myocardium. The profoundly low local glucose concentration in myocardium and skeletal muscle during endotoxemic shock with preservation of the lactate to pyruvate ratios suggests lactate utilization and/or differences in the Krebs cycle. Another interesting finding was the ability of skeletal muscle to preserve the lactate to pyruvate ratio during endotoxemic but not hemorrhagic shock, reflecting again the different lactate fates and perhaps the different mitochondrial densities between myocardium and skeletal muscle.

Sepsis induces significant changes in myocardial metabolism, including a reduction in the oxygen extraction ratio of the myocardium [2,3] and a shift in metabolic substrate from using free fatty acids to increased utilization of lactate. To understand the differences in myocardial and skeletal muscle metabolism observed by Chew and colleagues [1], we explore changes in substrate metabolism observed during a septic inflammatory response.

Sepsis is unique amongst types of shock in that it is the result of a complex interaction between the infecting microorganism and the host immune, inflammatory and coagulation responses. The host innate immune response is triggered through interaction of pathogen molecules with innate immune receptors with subsequent release of pro- and anti-inflammatory cytokines, stimulation of adaptive immunity, and activation of the coagulation system. Recent data suggest that septic shock may have unique effects on substrate utilization with accelerated glucose metabolism, despite compble pyruvate and lactate levels [1]. Reversible cardiomyocyte hypocontractility also occurs, possibly related to hibernation in order to maintain myocyte viability by limiting oxygen consumption, energy requirements and ATP. Whether a direct metabolic link connecting metabolic substrates and contractility exists remains to be demonstrated. It is notable, however, that Chew and colleagues [1] observed a significant drop in ejection fraction and impaired ventricular relaxation.

Cardiomyocytes possess the ability to act as substrate 'omnivores', changing their energy substrate in response to demand, ischemia and inflammatory stimuli. Prior studies have demonstrated the alteration in oxidative phosphorylation that occurs within mitochondria during sepsis, despite adequate oxygen availability and the preservation of ATP [4]. This also occurs during ischemia, likely from a different mechanism with intracellular ATP maintained by increased aerobic glycolysis. Concurrently, glucose transporters GLUT1 and GLUT4 increase glucose uptake with glycogen deposition in the cells [5].

The change in myocardial metabolism is not unique, but also is a function of the immune system whereby immune cells must switch from a resting quiescent state to an active state. Accelerated rates of glycolysis can occur through lipopolysaccharide activation of macrophages and dendritic cells through Toll-like receptor 4 (TLR4) in M1 inflammatory macrophages and T-helper 17 lymphocytes [6,7]. On the other hand, cells that limit inflammation, such as regulatory T cells, M2 anti-inflammatory macrophages and quiescent memory T cells that carry the CD8 antigen, exhibit oxidative metabolism with more limited rates of glycolysis [8,9]. This process is very energy demanding and it has been shown that activated T cells can increase glucose uptake 20- to 40-fold in preption to divide [10]. Amino acid and lipid metabolism is suppressed in order to permit cell expansion and hexokinase activity is increased, an enzyme involved in both glycolysis and the catabolic pentose phosphate pathway [11]. Free fatty acids are also activators of NF-κB through TLR4 signaling in adipocytes and skeletal muscle, and may have a similar effect in the myocardium [12]. Therefore, aerobic glycolysis is required for immune activation of macrophages, dendritic cells and T-cell effectors, but has little effect on cell proliferation or survival [6]. In activated T cells, this process may be regulated by GADPH binding to AU-rich elements in interferon-γ mRNA, thereby controlling its cytokine translation [13].

Cardiomyocytes share a number of features analogous to the antigen-presenting cells, including expression of TLRs [14,15]. In response to TLR activation, cardiomyocytes express many pro- and anti-inflammatory molecules, including cytokines (for example, tumor necrosis factor-α, interleukin-1β), chemokines, cell surface adhesions molecules, triggering of apoptotic pathways and increased expression of calcium channel binding proteins. Intercellular adhesion molecule 1 binds the cardiomyocyte cytoskeleton, disrupting normal intracellular calcium release and resulting in decreased contractility [16]. Calcium channel binding proteins S100A8 and S100A9 bind to the sarcoplasmic reticulum calcium channel SERCA2, further decreasing contractility [17]. Lastly, extracellular heat shock protein 70 acts through TLR2 to activate NF-κB and decrease contractility [18]. Therefore, it is not surprising that the changes in myocardial metabolism observed during the septic inflammatory response may share features with changes in metabolism observed in inflammatory cells. It is conceivable that changes in substrate metabolism observed in cardiomyocytes may be due to mechanisms shared with other inflammatory cells. Whether the myocardium decreases contractility to reflect the relatively inefficient process of aerobic glycolysis remains to be solved.

### Abbreviations

NF: nuclear factor; TLR: Tol-like receptor.

### Competing interests

The authors declare that they have no competing interests.
